# Impact and attribute of each obesity-related cardiovascular risk factor in combination with abdominal obesity on total health expenditures in adult Japanese National Health insurance beneficiaries: The Ibaraki Prefectural health study

**DOI:** 10.1016/j.je.2016.08.009

**Published:** 2017-03-01

**Authors:** Toshimi Sairenchi, Hiroyasu Iso, Kazumasa Yamagishi, Fujiko Irie, Masanori Nagao, Mitsumasa Umesawa, Yasuo Haruyama, Gen Kobashi, Hiroshi Watanabe, Hitoshi Ota

**Affiliations:** a Department of Public Health, Dokkyo Medical University School of Medicine, Shimotsugagun-Mibu, Japan; b Ibaraki Health Plaza, Mito, Japan; c Ibaraki Health Service Association, Mito, Japan; d Public Health, Department of Social Medicine, Osaka University Graduate School of Medicine, Osaka, Japan; e Department of Public Health Medicine, Faculty of Medicine, University of Tsukuba, Tsukuba, Japan; f Department of Health and Welfare, Ibaraki Prefectural Office, Mito, Japan

**Keywords:** Abdominal obesity, Cardiovascular disease, Cohort study, Health expenditure, Risk factor

## Abstract

**Background:**

The aim of this study was to examine the attribution of each cardiovascular risk factor in combination with abdominal obesity (AO) on Japanese health expenditures.

**Methods:**

The health insurance claims of 43,469 National Health Insurance beneficiaries aged 40–75 years in Ibaraki, Japan, from the second cohort of the Ibaraki Prefectural Health Study were followed-up from 2009 through 2013. Multivariable health expenditure ratios (HERs) of diabetes mellitus (DM), high low-density lipoprotein cholesterol (LDL-C), low high-density lipoprotein cholesterol (HDL-C), and hypertension with and without AO were calculated with reference to no risk factors using a Tweedie regression model.

**Results:**

Without AO, HERs were 1.58 for DM, 1.06 for high LDL-C, 1.27 for low HDL-C, and 1.31 for hypertension (all *P* < 0.05). With AO, HERs were 1.15 for AO, 1.42 for DM, 1.03 for high LDL-C, 1.11 for low HDL-C, and 1.26 for hypertension (all *P* < 0.05, except high LDL-C). Without AO, population attributable fractions (PAFs) were 2.8% for DM, 0.8% for high LDL-C, 0.7% for low HDL-C, and 6.5% for hypertension. With AO, PAFs were 1.0% for AO, 2.3% for DM, 0.4% for low HDL-C, and 5.0% for hypertension.

**Conclusions:**

Of the obesity-related cardiovascular risk factors, hypertension, independent of AO, appears to impose the greatest burden on Japanese health expenditures.

## Highlights

• The highest population attributable fraction was for hypertension without obesity.
• The total population attributable fraction of hypertension was 11.7%.
• Hypertension could impose the greatest attribute on Japanese health expenditures.

## Introduction

Ratios of total health expenditures to Gross Domestic Product have been increasing in most Organization for Economic Co-operation and Development (OECD) countries.^1^ Many countries have health care systems that can be divided into three categories: (i) social insurance systems, such as in Japan, France, and Germany; (ii) tax-based systems, such as in the United Kingdom (UK) and Sweden; and (iii) limited services to the elderly or disabled in the United States of America (USA), that is Medicare and Medicaid.^2^ The proportion of population coverage reached 100% in 22 of 31 OECD countries. Moreover, the coverage in the USA is being expanded by the Affordable Care Act.^3^

Cardiovascular diseases, such as ischemic heart diseases and cerebrovascular diseases, result in high mortality in most OECD countries,^1^ which can lead to high expenditures. Many previous studies have shown the associations of abdominal obesity, diabetes mellitus, high serum total cholesterol levels, low high-density lipoprotein cholesterol (HDL-C) levels, and hypertension, which are major risk factors for cardiovascular risk factors in terms of obesity and metabolic syndrome, with health expenditures.^4^, ^5^, ^6^, ^7^, ^8^, ^9^, ^10^, ^11^, ^12^, ^13^, ^14^ Several studies have shown that metabolic syndrome per se and abdominal obesity might not have an important effect on health expenditures in the USA and Taiwan.^4^, ^5^

However, to the best of our knowledge, no study has investigated the relationships of each cardiovascular risk factor in combination with abdominal obesity and their effects on health expenditures. Thus, the aim of the present study was to examine the impact, as indicated by the health expenditure ratio (HER), and attribution, as indicated by the population attributable fraction (PAF), of cardiovascular risk factors, with or without abdominal obesity, on health expenditures in Japan.

## Methods

### Health insurance system in Japan

The Japanese government has organized several health insurance schemes,^15^ and all citizens are obliged to join one. The National Health Insurance, one of them, is mainly for farmers, self-employed persons, retired persons, and their nonworking dependents aged less than 75 years. The Late-Stage Medical Care System for the Elderly is mainly for persons aged 75 years or more. Access to medical care is unlimited for all persons. The unit prices for medical care are decided by the government. Individuals must pay 10–30% of the medical fees, and the remainder is paid by the insurance. In the present study, the health expenditures included the portion of medical expenses paid individually.

### Study cohort and population

A total of 90,442 beneficiaries aged 40–75 years who completed a health check-up conducted by 21 of 44 Japanese National Health Insurance schemes in Ibaraki Prefecture in fiscal year 2009 (from April 2009 to March 2010) were recruited in the second cohort of the Ibaraki Prefectural Health Study. Written informed consent was obtained from 58,757 of those beneficiaries. However, 5418 individuals could not be matched with their health check-up data because of invalid numbering. Thus, the second cohort of the Ibaraki Prefectural Health Study consisted of 53,339 participants. The participants' health claim records were followed-up until the end of fiscal year 2013.

In this cohort study, 4550 participants with missing values for waist circumference, both fasting blood glucose and glycohemoglobin A1c (HbA1c) levels, low-density lipoprotein cholesterol (LDL-C) levels, HDL-C levels, systolic blood pressure (SBP), diastolic blood pressure (SBP), age, sex, smoking status, drinking habits, proteinuria, and/or of the follow-up period were excluded. Furthermore, 5320 participants with a history of stroke, heart disease, and/or kidney disease were excluded. Thus, the data of 43,469 participants were analyzed.

### Baseline measurements

At baseline, the subjects completed a self-administered lifestyle questionnaire. The questionnaire included questions about smoking status and drinking habits.

The health check-ups were conducted by the Ibaraki Health Service Association for the participants in 20 districts and by the Hitachi Medical Center for the participants in one district. Most (96%) of the participants underwent the health check-ups by the Ibaraki Health Service Association.

For the check-ups conducted by the Ibaraki Health Service Association, waist circumferences were measured in light clothing. SBP and DBP were measured using the right arm of seated subjects by trained observers using automated sphygmomanometers. Blood samples were drawn from seated subjects; fasting was not required. Plasma glucose levels were measured using an enzymatic method if the subjects were fasting. HbA1c levels were measured using immunoassay if the subjects were non-fasting or had unknown fasting status. In this study, the HbA1c levels are shown according to the National Glycohemoglobin Standardization Program. LDL-C levels were measured using an enzymatic method. Proteinuria levels were tested using a dipstick, and trace positive samples of proteinuria were re-examined using a sulphosalicylic acid test. An interview was conducted to ascertain the use of anti-diabetic medication, anti-dyslipidemic medication, and anti-hypertension medication, as well as histories of stroke, heart disease, and kidney disease.

For the check-ups conducted by the Hitachi Medical Center, HbA1c levels were measured using a high-performance liquid chromatography method if the subjects were non-fasting or had unknown fasting status. However, both laboratories participated in the same quality control programs directed by the Japan Medical Association, the National Federation of Industrial Health Organization, and the Ibaraki Association of Medical Technologists. Other measurement methods were the same as for check-ups conducted by the Ibaraki Health Service Association.

### Follow-up surveillance

To ascertain the medical expenditures of the participants, medical (outpatient and inpatient), dental, and pharmaceutical health insurance claim records of the participants were obtained from the Ibaraki National Health Insurance Organization. Moreover, the dates of acquisition and loss of eligibility of a recipient were obtained to ascertain the days the participants were eligible recipients. Follow-ups were restarted if the participants who had lost their eligibility to be recipients re-acquired their eligibility to be recipients. In addition, the follow-ups were continued even if the participants changed from the National Health Insurance to the Late-Stage Medical Care System for the Elderly.

### Definitions

Abdominal obesity was defined as waist circumference ≥85 cm for men and ≥90 cm for women, referring to the Japanese definition of metabolic syndrome.^16^ Diabetes was defined as fasting blood glucose ≥126 mg/dL (≥7.0 mmol/L), HbA1c ≥ 6.5%, or anti-diabetic medication use according to the guidelines of the World Health Organization.^17^, ^18^ High LDL-C was defined as LDL-C ≥ 160 mg/dL (≥4.1 mmol/L) or anti-dyslipidemic medication use, and low HDL-C was defined as HDL-C < 40 mg/dL (<1.0 mmol/L) according to the executive summary of the Third Report of the National Cholesterol Education Program Expert Panel on Detection, Evaluation, and Treatment of High Blood Cholesterol in Adults III.^19^ Hypertension was defined as systolic blood pressure (SBP) ≥140 mm Hg, DBP ≥90 mm Hg, or anti-hypertensive medication use according to the 2014 Evidence-Based Guideline for the Management of High Blood Pressure in Adults Report from the Panel Members Appointed to the Eighth Joint National Committee.^20^ Smoking habit was divided into three categories: non-smoker, ex-smoker, and current smoker. Alcohol intake was divided into seven categories: non-drinker, ex-drinker, 1–3 days per month, 1–2 days per week, 3–4 days per week, 5–6 days per week, and every day. Proteinuria was divided into four categories: −, +, 2+, and 3+.

### Statistical analysis

The unit of health expenditures was set as the Japanese yen (i.e., 123.9 Japanese yen = 1 USA dollar, at the exchange rates published on June 17, 2015). The *P* values for differences in baseline characteristics according to the presence or absence of each risk factor were calculated using analysis of variance for age, and using χ^2^ tests for sex, smoking status, drinking habits, and proteinuria.

HERs and 95% confidence interval (CI) according to the presence or absence of the risk factors were calculated with reference to no risk factor using a generalized linear model with an assumption of the Tweedie (compound Poisson-Gamma) distribution, which is the most widely used method in insurance claims modeling.^21^ The link function was logarithmic. The log-transformed years of eligibility to be a recipient were treated as an offset variable. Covariates included age and sex in the age- and sex-adjusted model. Covariates included age, sex, smoking status, drinking habits, proteinuria, and the risk factors of interest in the multivariable model. All statistical tests were 2-sided, and *P* < 0.05 was regarded as significant. The statistical analyses were conducted with SAS, version 9.4 (SAS Institute, Inc, Cary, NC, USA). Furthermore, PAFs were calculated using the following formula:

$$\text{PAF}=pd\left(\frac{mHER-1}{mHER}\right)\text{,}$$

where *pd* is the proportion of expenditures from the group exposed to the risk factor among total expenditures, and *mHER* is a multivariable HER.^22^ The 95% confidence intervals of PAF were calculated using the following formula^23^:

Θ=log(1−PAF),

$$Var\left(\Theta \right)=\frac{PAF^{2}}{{\left(1-PAF\right)}^{2}}\left(\frac{\hat{V}}{{\left(mHER-1\right)}^{2}}+\frac{2}{e_{1}\left(mHER-1\right)}+\frac{e_{2}}{e_{1}\left(e_{2}+e_{2}\right)}\right)\text{,}$$

where $\hat{V}$ is a variance estimate for log(mHER), $e_{1}$ is expenditures from exposed persons, and $e_{2}$ is the expenditures from non-exposed persons.

$$CI_{PAF}=1-exp\left(\Theta \pm 1.96\sqrt{Var\left(\Theta \right)}\right)\text{,}$$

where $CI_{PAF}$ is a 95% confidence interval for PAF.

### Standard protocol approvals, registrations, and patient consent

Written informed consent was obtained from all individual participants included in the study. The Ethics Committee of Ibaraki Prefecture and the Bioethics Committee of Dokkyo Medical University approved this study.

## Results

During 209,101 person-years of follow-up for 43,469 participants, visits to clinics and/or hospitals of 43,169 persons, 3,870,460 claims, and health expenditures of 59,991,370,680 Japanese yen ($484,191,854 USA dollars) were observed. Of the participants, 158 men and 142 women had health expenditures of zero Japanese yen.

The baseline characteristics are shown in Table 1. Mean age and the proportions of men, smoking status, drinking habits, and proteinuria were significantly different according to the presence or absence of abdominal obesity, diabetes mellitus, high LDL-C, low HDL-C, and hypertension, excluding proteinuria with high LDL-C.

**Table 1:** Baseline characteristics according to absence or presence of cardiovascular risk factors among 43,469 Japanese National Health Insurance beneficiaries aged 40–74 years in Ibaraki, Japan, 2009.

	Abdominal obesity		*P* for difference^a^	Diabetes mellitus		*P* for difference^a^	High LDL-C		*P* for difference^a^	Low HDL-C		*P* for difference^a^	Hypertension		*P* for difference^a^
	Absence	Presence		Absence	Presence		Absence	Presence		Absence	Presence		Absence	Presence	
Number of participants	29,383	14,086		39,345	4124		33,681	9788		40,804	2665		25,645	17,824	
Age, years, mean (SD)	62.9 (8.2)	63.7 (8.2)	<0.001	62.9 (8.3)	65.5 (6.5)	<0.001	62.8 (8.5)	64.5 (7.0)	<0.001	63.1 (8.2)	64.0 (8.5)	<0.001	61.4 (8.8)	65.7 (6.6)	<0.001
Male, n (%)	9633 (32.8)	9188 (65.2)	<0.001	16,398 (41.7)	2423 (58.8)	<0.001	15,977 (47.4)	2844 (29.1)	<0.001	16,833 (41.3)	1988 (74.6)	<0.001	10,279 (40.1)	8542 (47.9)	<0.001
Smoking status, n (%)			<0.001			<0.001			<0.001			<0.001			<0.001
Non-smoker	23,009 (78.3)	9491 (67.4)		29,624 (75.3)	2876 (69.7)		24,493 (72.7)	8007 (81.8)		30,968 (75.9)	1532 (57.5)		18,833 (73.4)	13,667 (76.7)	
Ex-smoker	2346 (8.0)	1806 (12.8)		3666 (9.3)	486 (11.8)		3433 (10.2)	719 (7.3)		3834 (9.4)	318 (11.9)		2407 (9.4)	1745 (9.8)	
Current smoker	4028 (13.7)	2789 (19.8)		6055 (15.4)	762 (18.5)		5755 (17.1)	1062 (10.9)		6002 (14.7)	815 (30.6)		4405 (17.2)	2412 (13.5)	
Drinking habits, n (%)			<0.001			<0.001			<0.001			<0.001			<0.001
Non-drinker	16,417 (55.9)	5765 (40.9)		20,181 (51.3)	2001 (48.5)		16,104 (47.8)	6078 (62.1)		20,823 (51.0)	1359 (51.0)		13,767 (53.7)	8415 (47.2)	
Ex-drinker	468 (1.6)	326 (2.3)		656 (1.7)	138 (3.3)		648 (1.9)	146 (1.5)		668 (1.6)	126 (4.7)		476 (1.9)	318 (1.8)	
1–3 days per month	2405 (8.2)	1044 (7.4)		3089 (7.9)	360 (8.7)		2615 (7.8)	834 (8.5)		3182 (7.8)	267 (10.0)		2222 (8.7)	1227 (6.9)	
1–2 days per week	2016 (6.9)	993 (7.0)		2740 (7.0)	269 (6.5)		2292 (6.8)	717 (7.3)		2808 (6.9)	201 (7.5)		1873 (7.3)	1136 (6.4)	
3–4 days per week	1806 (6.1)	1060 (7.5)		2605 (6.6)	261 (6.3)		2358 (7.0)	508 (5.2)		2676 (6.6)	190 (7.1)		1665 (6.5)	1201 (6.7)	
5–6 days per week	1835 (6.2)	1266 (9.0)		2803 (7.1)	298 (7.2)		2618 (7.8)	483 (4.9)		2939 (7.2)	162 (6.1)		1657 (6.5)	1444 (8.1)	
Everyday	4436 (15.1)	3632 (25.8)		7271 (18.5)	797 (19.3)		7046 (20.9)	1022 (10.4)		7708 (18.9)	360 (13.5)		3985 (15.5)	4083 (22.9)	
Proteinuria, n (%)												0.077			<0.001
	28,976 (98.6)	13,588 (96.5)		38,745 (98.5)	3819 (92.6)		33,012 (98.0)	9552 (97.6)		40,009 (98.1)	2555 (95.9)		25,403 (99.1)	17,161 (96.3)	
+	301 (1.0)	343 (2.4)		442 (1.1)	202 (4.9)		474 (1.4)	170 (1.7)		572 (1.4)	72 (2.7)		183 (0.7)	461 (2.6)	
2+	82 (0.3)	120 (0.9)		128 (0.3)	74 (1.8)		151 (0.4)	51 (0.5)		173 (0.4)	29 (1.1)		50 (0.2)	152 (0.9)	
3+	24 (0.1)	35 (0.2)		30 (0.1)	29 (0.7)		44 (0.1)	15 (0.2)		50 (0.1)	9 (0.3)		9 (0.0)	50 (0.3)	

LDL-C, low-density lipoprotein cholesterol; HDL-C, high-density lipoprotein cholesterol; SD, standard deviation.

a Tested by analysis of variance for age and a chi-square test for other variables.

The HERs and PAFs according to the presence or absence of each risk factor for the participants are shown in Table 2. Significant associations between each risk factor and health expenditures were found in the age- and sex-adjusted analyses, as well as in the multivariable analyses. The highest multivariable HER was for diabetes mellitus. On the other hand, the highest PAF was observed for hypertension.

**Table 2:** Health expenditure ratios and population attributable fractions according to the presence or absence of each risk factor among 43,469 Japanese National Health Insurance beneficiaries aged 40–74 years in Ibaraki, Japan, 2009–2013.

Risk factors at baseline	Number of participants	Person-years	Health expenditures per person-year, JPY (USD)	Sex- and age-adjusted health expenditure ratio^a^ (95% CI)	Multivariable health expenditure ratio^b^ (95% CI)	Population attributable fraction (%) (95% CI)
No risk	14,258	68,031	200,297 (1617)	–	1 (ref)	
Abdominal obesity	14,086	67,789	335,177 (2705)	1.20 (1.17,1.22)	1.10 (1.07,1.12)	3.3 (2.6, 4.0)
Diabetes mellitus	4124	19,825	467,634 (3774)	1.61 (1.57,1.66)	1.50 (1.46,1.55)	5.2 (4.9, 5.5)
High LDL-C	9788	47,355	315,555 (2547)	1.10 (1.07,1.12)	1.05 (1.03,1.07)	1.1 (0.6, 1.6)
Low HDL-C	2665	12,726	376,858 (3042)	1.26 (1.22,1.31)	1.18 (1.14,1.22)	1.2 (1.0, 1.5)
Hypertension	17,824	86,387	362,345 (2924)	1.33 (1.31,1.36)	1.29 (1.27,1.31)	11.7 (11.0, 12.5)

CI, confidence interval; HDL-C, high-density lipoprotein cholesterol; JPY, Japanese yen; LDL-C, low-density lipoprotein cholesterol; USD, United States dollar.

a The reference category was the absence of each risk factor.

b Adjusted for age, sex, smoking status (non-smoker, ex-smoker, and current smoker), drinking habits (non-drinker, ex-drinker, 1–3 days per month, 1–2 days per week, 3–4 days per week, 5–6 days per week, and every day), proteinuria (−, +, 2+, and 3+), and risk factors in this table with each other.

The multivariable HERs and PAFs according to the combination of risk factors and abdominal obesity are shown in Table 3. Significantly higher multivariable HERs were observed for all combinations, except for high LDL-C with abdominal obesity. The highest multivariable HER was observed for diabetes mellitus without abdominal obesity. The highest PAF was observed for hypertension without abdominal obesity followed by hypertension with abdominal obesity.

**Table 3:** Multivariable health expenditure ratios and population attributable fractions according to combinations of risk factors and abdominal obesity among 43,469 Japanese National Health Insurance beneficiaries aged 40–74 years in Ibaraki, Japan, 2009–2013.

Risk factors at baseline	Number of participants	Person-years	Health expenditures per person-year, JPY (USD)	Multivariable health expenditure ratio^a^ (95% CI)	Population attributable fraction (%) (95% CI)
Without abdominal obesity					
No risk	14,258	6,8031	200,297 (1617)	1 (ref)	–
Diabetes mellitus	2054	9912	459,062 (3705)	1.58 (1.51,1.64)	2.8 (2.6, 3.0)
High LDL-C	6320	30,637	294,879 (2380)	1.06 (1.03,1.09)	0.8 (0.4, 1.2)
Low HDL-C	1148	5512	368,911 (2977)	1.27 (1.20,1.34)	0.7 (0.6, 0.9)
Hypertension	10,074	48,855	342,034 (2761)	1.31 (1.28,1.34)	6.5 (6.0, 7.0)
With abdominal obesity					
No risk (Only abdominal obesity)	3858	18,460	240,777 (1943)	1.15 (1.11,1.18)	1.0 (0.8, 1.2)
Diabetes mellitus	2070	9913	476,205 (3843)	1.42 (1.36,1.48)	2.3 (2.1, 2.6)
High LDL-C	3468	16,718	353,443 (2853)	1.03 (0.99,1.06)	–
Low HDL-C	1517	7214	382,929 (3019)	1.11 (1.05,1.16)	0.4 (0.2, 0.7)
Hypertension	7750	37,532	388,784 (3138)	1.26 (1.22,1.30)	5.0 (4.4, 5.6)

CI, confidence interval; HDL-C, high-density lipoprotein cholesterol; JPY, Japanese yen; LDL-C, low-density lipoprotein cholesterol; USD, United States dollar.

a Adjusted for age, sex, smoking status (non-smoker, ex-smoker, and current smoker), drinking habits (non-drinker, ex-drinker, 1–3 days per month, 1–2 days per week, 3–4 days per week, 5–6 days per week, and every day), proteinuria (−, +, 2+, and 3+), and risk factors in this table with each other.

## Discussion

To the best of our knowledge, the present study is the first to show that the obesity-related cardiovascular risk factor related to the greatest attribution on the Japanese National Health Insurance expenditures was hypertension, independent of abdominal obesity. The present results support the importance of the prevention of hypertension for persons with and without abdominal obesity for health expenditures in Japan. In addition, metabolic syndrome, which assumes abdominal obesity to be indispensable, might have to be treated as one of multiple risk factors from the perspective of primary prevention for a sustainable health insurance system.

Several studies have investigated the relationships between cardiovascular risk factors and health expenditures.^4^, ^5^, ^6^, ^7^, ^8^, ^9^, ^10^, ^11^, ^12^, ^13^, ^14^ The Cardiovascular Health Study^5^ showed higher costs of 14.9% (95% CI, 4.3%–26.7%) for abdominal obesity, 15.8% (95% CI, 1.7%–31.8%) for low HDL cholesterol, and 20.4% (95% CI, 10.1%–31.7%) for elevated blood pressure of the total Medicare costs. The study also showed that there was no significant association between metabolic syndrome and Medicare costs. The Chicago Heart Association Detection Project in Industry^6^ showed an association between high blood pressure (SBP ≥120 mm Hg or DBP ≥80 mm Hg) and Medicare costs related to cardiovascular disease in men and women. In another report of the Chicago Heart Association Detection Project in Industry,^7^ the adjusted average annual total Medicare costs of subjects with body mass index (BMI) 25.0–29.9 kg/m^2^ compared with BMI 18.5–24.9 kg/m^2^ were 1.12-fold higher in men and 1.20-fold higher in women. A large prospective cohort study^8^ showed that the average total Medicare expenditures of beneficiaries with diabetes was approximately 1.7-fold higher than that of beneficiaries without diabetes.

The Elderly Nutrition and Health Survey in Taiwan^4^ showed adjusted cost ratios of 1.45 for high fasting glucose (≥100 mg/dL or current use of diabetes medication) and 1.46 for high blood pressure (SPB ≥130 mm Hg, DBP ≥85 mm Hg, or current use of antihypertensive medication) in men, whereas central obesity (waist circumference ≥90 cm for men or ≥80 cm for women) was not significant. The Ohsaki Study^9^ showed that the total medical costs of participants with BMI 25.0–29.9 kg/m^2^ and with BMI ≥30.0 kg/m^2^ were approximately 1.1- and 1.2-fold higher, respectively, than participants with BMI 21.0–22.9 kg/m^2^, and the excess direct costs attributable to BMI ≥25.0 kg/m^2^ were 3.2% of total health care expenditures. The subgroup analysis of the Ohsaki Study^13^ indicated that the excess adjusted total cost was 9.4% for overweight/obesity (BMI ≥25 kg/m^2^), 35.6% for hypertension (SBP ≥140 mm Hg, DBP ≥90 mm Hg, or antihypertensive medication use), 42.2% for hyperglycemia (casual plasma glucose ≥150 mg/dL or history of diabetes), and no excess cost for dyslipidemia (serum total cholesterol ≥220 mg/dL and serum high-density lipoprotein cholesterol <40 mg/dL). The Health Promotion Research Committee of the Shiga National Health Insurance Organizations^12^ reported that subjects with BMI ≥25.0 kg/m^2^ had approximately 1.3-fold higher adjusted geometric mean medical expenditures compared with those with BMI of 18.5–24.9 kg/m^2^, and the excess medical costs attributable to obesity (BMI ≥25.0 kg/m^2^) were 3.1% of total medical costs. The Health Promotion Research Committee of the Shiga National Health Insurance Organizations^10^ also showed that the adjusted geometric mean of beneficiaries with Stage 1 hypertension compared to those with normal blood pressure was 1.24-fold higher for men and 1.01-fold higher for women, and they also showed that the percentage of stage 1 and 2 hypertension-related medical costs for the beneficiaries was 14.2%. Another report of the Health Promotion Research Committee of the Shiga National Health Insurance Organizations^11^ showed that total medical expenditure was approximately two-fold higher for subjects with diabetes (history of diabetes) than for those without diabetes. Furthermore, in another report of the Health Promotion Research Committee of the Shiga National Health Insurance Organizations,^14^ the excess medical expenditures attributable to cardiovascular risk factors with normal weight were higher than in those who were overweight (BMI ≥25.0 kg/m^2^). Those previous studies suggested that each cardiovascular risk factor (i.e., obesity, hyperglycemia, dyslipidemia, and high blood pressures) was associated with health expenditures, as in the present study. However, those previous studies did not investigate the association of each cardiovascular risk factor with health expenditures in combination with abdominal obesity.

The strength of the present study was the statistical analysis with a Tweedie distribution, which is the most widely used mixture distribution model in insurance claims modeling. The Tweedie distribution is defined as a Poisson sum of gamma random variables, known as an *aggregate loss* in actuarial science.^21^ The Tweedie model is a generalized linear model from the exponential family. A Tweedie random variable can be represented as a Poisson sum of gamma distributed random variables. That is,

$$Y=\sum_{i=1}^{N}Y_{i}$$

where *N* has a Poisson distribution, and $Y_{i}$ s have independent, identical gamma distributions. In this case, *Y* has a discrete mass at 0, Pr(Y=0)=Pr(N=0)=exp(−λ), where *λ* is the mean of Poisson distribution, and the probability density of *Y* f(y) is represented by an infinite series fory>0.^24^ That is, a Tweedie distribution can treat a gamma distribution with inflated zero data, such as health claim data, simultaneously. In Tweedie distributions, a compound Poisson-Gamma distribution is produced when the power parameter *p* is >1 and <2. In the present study, all power parameters estimated were approximately 1.8 (data not shown) which indicated that the distributions were valid.

On the other hand, the present study has several limitations. First, the definition of abdominal obesity did not refer to the National Cholesterol Education Program Expert Panel on Detection, Evaluation, and Treatment of High Blood Cholesterol in Adults III criteria.^19^ However, the Japanese criteria of waist circumference^16^ have been adopted on a nationwide scale in Japan. For instance, the Japanese Society of Hypertension Guidelines for the Management of Hypertension adopted the Japanese waist circumference criteria (≥85 cm for men and ≥90 cm for women) as defining abdominal obesity. According to the National Health and Nutrition Survey in Japan, 2013,^25^ conducted by the Ministry of Health, Labour and Welfare, Japan, the prevalence of high waist circumference (≥85 cm for men and ≥90 cm for women) was 34% among a representative sample of the Japanese population aged 20 years or older. Second, further studies are warranted to ascertain the generalizability of the findings due to the low participation rate (34%) in health-checkups in the study area and the low proportion of study subjects among participants in health-checkups.

In summary, hypertension, independent of abdominal obesity, appears to impose the greatest burden among the obesity-related cardiovascular risk factors on Japanese National Health Insurance expenditures.

## Conflict of interest

None declared.

## References

[1] World Health Organization (2014). World Health Statistics 2014.

[2] Sasaki T., Izawa M., Okada Y. (2015). Current trends in health insurance systems: OECD countries vs. Japan. Neurol Med Chir (Tokyo).

[3] Diebel N.D. (2015). US health care system: we can do better. Am J Obstet Gynecol.

[4] Chang Y.H., Chen R.C., Lee M.S., Wahlqvist M.L. (2012). Increased medical costs in elders with the metabolic syndrome are most evident with hospitalization of men. Gend Med.

[5] Curtis L.H., Hammill B.G., Bethel M.A., Anstrom K.J., Gottdiener J.S., Schulman K.A. (2007). Costs of the metabolic syndrome in elderly individuals: findings from the Cardiovascular Health Study. Diabetes Care.

[6] Daviglus M.L., Liu K., Greenland P. (1998). Benefit of a favorable cardiovascular risk-factor profile in middle age with respect to Medicare costs. N Engl J Med.

[7] Daviglus M.L., Liu K., Yan L.L. (2004). Relation of body mass index in young adulthood and middle age to Medicare expenditures in older age. JAMA.

[8] Krop J.S., Saudek C.D., Weller W.E., Powe N.R., Shaffer T., Anderson G.F. (1999). Predicting expenditures for Medicare beneficiaries with diabetes. A prospective cohort study from 1994 to 1996. Diabetes Care.

[9] Kuriyama S., Tsuji I., Ohkubo T. (2002). Medical care expenditure associated with body mass index in Japan: the Ohsaki Study. Int J Obes Relat Metab Disord.

[10] Nakamura K., Okamura T., Kanda H. (2005). Impact of hypertension on medical economics: a 10-year follow-up study of national health insurance in Shiga. Jpn Hypertens Res.

[11] Nakamura K., Okamura T., Kanda H. (2008). Medical expenditure for diabetic patients: a 10-year follow-up study of National Health Insurance in Shiga, Japan. Public Health.

[12] Nakamura K., Okamura T., Kanda H. (2007). Medical costs of obese Japanese: a 10-year follow-up study of National Health Insurance in Shiga, Japan. Eur J Public Health.

[13] Ohmori-Matsuda K., Kuriyama S., Hozawa A., Nakaya N., Shimazu T., Tsuji I. (2007). The joint impact of cardiovascular risk factors upon medical costs. Prev Med.

[14] Okamura T., Nakamura K., Kanda H. (2007). Effect of combined cardiovascular risk factors on individual and population medical expenditures: a 10-year cohort study of national health insurance in a Japanese population. Circ J.

[15] Annual Health (2015). Labour and Welfare Report 2013–2014 [homepage on the Internet]. http://www.mhlw.go.jp/english/wp/wp-hw8/index.html.

[16] Metabolic Syndrome Shindan Kijyun Kento Iinkai (2005). Definition and the diagnostic standard for metabolic syndrome–committee to evaluate diagnostic standards for metabolic syndrome. Nihon Naika Gakkai Zasshi.

[17] World Health Organization (2011). Use of Glycated Haemoglobin (HbA1c) in the Diagnosis of Diabetes Mellitus Abbreviated Report of a WHO Consultation.

[18] World Health Organization, International Diabetes Federation (2006). Definition and Diagnosis of Diabetes Mellitus and Intermediate Hyperglycemia: Report of a WHO/IDF Consultation.

[19] Expert Panel on Detection (2001). Evaluation, and treatment of high blood cholesterol in adults. Executive summary of the third report of the National Cholesterol Education Program (NCEP) expert panel on detection, evaluation, and treatment of high blood cholesterol in adults (Adult treatment panel III). JAMA.

[20] James P.A., Oparil S., Carter B.L. (2014). 2014 Evidence-based guideline for the management of high blood pressure in adults: report from the panel members appointed to the Eighth Joint National Committee (JNC 8). JAMA.

[21] Frees E.W. (2009). Regression Modeling with Actuarial and Financial Applications.

[22] Rockhill B., Newman B., Weinberg C. (1998). Use and misuse of population attributable fractions. Am J Public Health.

[23] Greenland S., Rothman K.J., Greenland S. (1998). Applications of stratified analysis methods. Modern Epidemiology.

[24] SAS Institute Inc (2015). SAS/STAT^®^ 14.1 User's Guide.

[25] Ministry of Health, Labour and Welfare, Japan (2015). The National Health and Nutrition Survey in Japan, 2013.

